# Discovery of novel isoflavone derivatives as AChE/BuChE dual-targeted inhibitors: synthesis, biological evaluation and molecular modelling

**DOI:** 10.1080/14756366.2017.1347163

**Published:** 2017-07-18

**Authors:** Bo Feng, Xinpeng Li, Jie Xia, Song Wu

**Affiliations:** a State Key Laboratory of Bioactive Substance and Function of Natural Medicines, Department of New Drug Research and Development, Institute of Materia Medica, Chinese Academy of Medical Sciences and Peking Union Medical College, Beijing100050, China;; b Food and Drug Administration of Beijing Yanqing District, Beijing102100, China

**Keywords:** Alzheimer’s disease, isoflavone derivatives, AChE/BuChE dual-targeted inhibitor, molecular modelling

## Abstract

AChE and BuChE are druggable targets for the discovery of anti-Alzheimer’s disease drugs, while dual-inhibition of these two targets seems to be more effective. In this study, we synthesised a series of novel isoflavone derivatives based on our hit compound G from *in silico* high-throughput screening and then tested their activities by *in vitro* AChE and BuChE bioassays. Most of the isoflavone derivatives displayed moderate inhibition against both AChE and BuChE. Among them, compound **16** was identified as a potent AChE/BuChE dual-targeted inhibitor (IC_50_: 4.60 μM for AChE; 5.92 μM for BuChE). Molecular modelling study indicated compound **16** may possess better pharmacokinetic properties, e.g. absorption, blood–brain barrier penetration and CYP2D6 binding. Taken together, our study has identified compound **16** as an excellent lead compound for the treatment of Alzheimer’s disease.

## Introduction

Alzheimer’s disease (AD) is one of the most common neurodegenerative diseases and accounts for more than 80% of dementia cases worldwide in elderly people^1^^,^^2^. It is in particular characterised by a forebrain cholinergic neuronal loss, a progressive cognitive decline and neuropsychiatric cholinergic disturbances, which seems to be closely related to pathological formation of β-amyloid^3^. Studies have demonstrated that memory impairment in patients with dementia is due to the selective and irreversible deficiency in the cholinergic functions^4^. Cholinesterase is the enzyme which catalyses the hydrolysis of acetylcholine, and thus inhibition of cholinesterase can be an effective way for the treatment of AD^5–7^.

**Figure 1.**
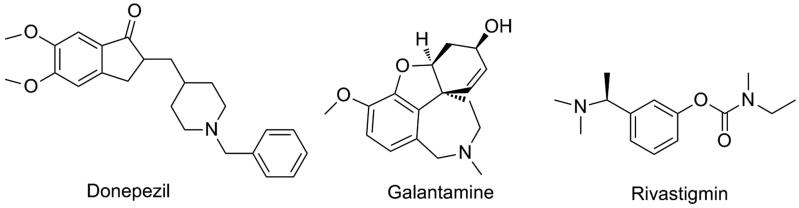
Structures of currently marketed cholinesterase inhibitors.

Two types of the cholinesterase, namely, acetylcholinesterase (AChE) and butyrylcholinesterase (BuChE) were discovered in the nervous system^8^. However, AChE has a 10^13^ fold higher acetylcholine hydrolytic activity than BuChE does under the same condition^9^^,^^10^. Many AChE inhibitors were discovered, of which donepezil^11^ and galantamine^12^ (cf. Figure 1) have been approved by Food and Drug Administration (FDA). Nevertheless, the clinical use demonstrated they have modest memorial and cognitive improving functions for patients with AD. Specifically, they only provide a symptomatic and palliative pharmacological effect instead of curing AD, and their effects would “wear off” after a certain period of treatment time^13^^,^^14^. Evidences suggested that additional inhibition of brain BuChE may become an important therapeutic strategy for AD treatment. It was reported that the BuChE had a key role that it could partly compensate for the action of AChE^15^. In addition, studies indicated that a balanced inhibition of both AChE and BuChE may be beneficial for treating the cognitive deficits^16^^,^^17^. Such multifunctional cholinestarase inhibitors have been identified so far and were showing positive clinical outcome for the treatment of AD^18^^,^^19^. For instance, the AChE/BuChE dual-targeted inhibitor, i.e. a “blockbuster drug” rivastigmin (cf. Figure 1) displayed a more potent effect than donepezil^20^.

**Figure 2.**
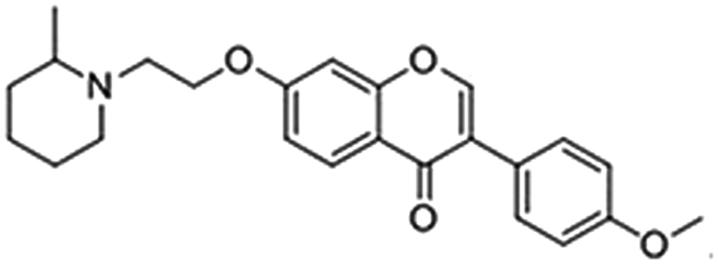
Chemical Structure of the hit compound **G**.

In order to identify new classes of AChE/BuChE dual-targeted inhibitors, *in silico* high-throughput screening (HTS) was performed to screen our in-house chemical library that contained more than 30,000 compounds. On the top-ranked compound list, an isoflavone derivative **G** (cf. Figure 2) attracted our attention as it displayed favourable binding modes with both AChE and BuChE (cf. Supplementary Figure S1). To the best of our knowledge, the scaffolds of flavonoids^21^^,^^22^ and its closely related ones such as homoisolavonoids^23^^,^^24^, trihydroxyflavone^25^^,^^26^ were privileged structures that showed beneficial effects on neurological disorders, e.g. anti-inflammation, metal chelation, neuroprotection, Aβ fibril formation inhibition and free radical scavenging effect. In addition, a recent study revealed a few compounds structurally similar to **G** were AChE inhibitors^21^. These evidences prompted us to test the activity of **G** against both AChE and BuChE. Interestingly, the compound **G** showed a dual-targeting effect, with an IC_50_ of 1.47 μM for AChE and 3.37 μM for BuChE. In this study, we explored its preliminary structure-activity relationship (SAR) by synthesising a series of novel isoflavone derivatives and testing their inhibitory effects on both AChE and BuChE. Then we carried out molecular docking to predict binding modes of isoflavone derivatives to AChE and BuChE. Moreover, we predicted the pharmacokinetic profile of those dual-targeting isoflavone derivatives, i.e. absorption, distribution, metabolism and excretion (ADME) properties.

## Materials and methods

### Chemistry

All melting points (mps) were measured by a Melting Point YRT-3 apparatus (Tianjin precision apparatus factory, China) and were uncorrected. NMR spectra were performed using 300 or 400 MHz spectrometers (Varian Mercury, USA) with TMS as an internal standard. High resolution mass spectra were determined by Thermo Scientific Exactive Plus mass spectrometry with ESI method (Thermo, USA). Some compounds were purified by medium-pressure preparative column chromatography (Shimadzu, Japan). The purity of all these synthesised compounds was determined by HPLC analysis (Waters, USA). All the materials were obtained from commercial suppliers and used without purification, unless otherwise specified. Yields were not optimised. TLC analysis was carried out on silica gel plates GF254 (Yantai chemical research institute, China). Column chromatography was performed on silica gel (200–300 mush; Qingdao Marine Chemical Inc.).

#### General procedure for the preparation of intermediates (a1–a4)

Formononetin (5 g, 18.6 mmol), finely grounded anhydrous K_2_CO_3_ (30 g, 270 mmol) and 1,2-dibromoethane (17.2 ml, 199 mmol) or 1,3-dibromopropane (20.1 ml, 199 mmol) or 1,4-dibromobutane (24 ml, 198 mmol) were added into 500 ml acetone solution. The mixture was refluxed for 10 h. The residues were then added into 100 ml water and stirred for 1 h and filtrated by suction. After that, the pastry was washed by 5 ml water for three times. The solid was finally dried in vacuum at 50 °C to give the desired product without further purification.

#### 7-(2-Bromoethoxy)-3-(4-methoxyphenyl)-4H-chromen-4-one (a1)

The compound was obtained as white solid in 93.4% yield. Purity 98.5% (by HPLC); mp 177.7–179.6 °C; ^1^H NMR (400 MHz, CDCl_3_) *δ* 8.23 (d, *J* = 8.8 Hz, 1H), 7.92 (s, 1H), 7.50 (d, *J* = 8.8 Hz, 2H), 7.02–6.96 (m, 3H), 6.86 (d, *J* = 2.0 Hz, 1H), 4.39 (t, *J* = 6.4 Hz, 2H), 3.84 (s, 3H), 3.69 (t, *J* = 6.4 Hz, 2H); ^13^C NMR (100 MHz, CDCl_3_) *δ* 175.9, 162.4, 159.8, 157.9, 152.2, 130.2, 128.2, 125.1, 124.2, 119.1, 114.7, 114.1, 101.2, 68.3, 55.5, 28.4. ESI-MS m/z 375.38 [M + H]^+^.

#### 7-(3-Bromopropoxy)-3–(4-methoxyphenyl)-4H-chromen-4-one (a2)

The compound was obtained as white solid in 91.5% yield. Purity 99.2% (by HPLC); mp 121.5–123.6 °C; ^1^H NMR (400 MHz, CDCl_3_) *δ* 8.22 (d, *J* = 8.8 Hz, 1H), 7.92 (s, 1H), 7.50 (d, *J* = 8.8 Hz, 2H), 7.02–6.91 (m, 3H), 6.87 (d, *J* = 2.4 Hz, 1H), 4.22 (t, *J* = 6.0 Hz, 2H), 3.84 (s, 3H), 3.63 (t, *J* = 6.0 Hz, 2H), 2.38 (m, *J* = 6.0 Hz, 2H); ^13^C NMR (100 MHz, CDCl_3_) *δ* 176.0, 163.1, 159.7, 158.0, 152.2, 130.3, 128.0, 125.1, 124.3, 118.7, 114.8, 114.1, 100.9, 66.1, 55.5, 32.1, 29.7. ESI-MS *m*/*z* 389.34 [M + H]^+^.

#### 7-(4-Bromobutoxy)-3-(4-methoxyphenyl)-4H-chromen-4-one (a3)

The compound was obtained as white solid in 88.6% yield. Purity 98.7% (by HPLC); mp 152.8–154.0 °C; ^1^H NMR (400 MHz, CDCl_3_) *δ* 8.21 (d, *J* = 8.8 Hz, 1H), 7.91 (s, 1H), 7.50 (d, *J* = 8.8 Hz, 2H), 6.99–6.96 (m, 3H), 6.84 (d, *J* = 2.4 Hz, 1H), 4.10 (t, *J* = 6.0 Hz, 2H), 3.84 (s, 3H), 3.51 (t, *J* = 6.0 Hz, 2H), 2.14–1.98 (m, 4H); ^13^C NMR (100 MHz, CDCl_3_) *δ* 175.8, 163.2, 159.6, 157.9, 152.0, 130.1, 127.8, 124.9, 124.2, 118.5, 114.7, 114.0, 100.6, 67.6, 55.3, 33.2, 29.3, 27.6. ESI-MS *m*/*z* 403.31 [M + H]^+^.

#### 7-(2-Bromoethoxy)-5-hydroxy-3-(4-methoxyphenyl)-4H-chromen-4-one (a4)

The procedure is the same as above method, however, the formononetin was replaced by 5-hydroxyl formononetin. The compound was obtained as white solid in 94.4% yield. Purity 99.6% (by HPLC); mp 157.1–159.2 °C; ^1^H NMR (400 MHz, CDCl_3_) *δ* 12.87 (s, 1H), 7.88 (s, 1H), 7.46 (d, *J* = 8.8 Hz, 2H), 6.98 (d, *J* = 8.8 Hz, 2H), 6.40 (dd, *J* = 2.0, 15.2 Hz, 2H), 4.36 (t, *J* = 6.0 Hz, 2H), 3.85 (s, 3H), 3.66 (t, *J* = 6.0 Hz, 2H); ^13^C NMR (100 MHz, CDCl_3_) *δ* 181.0, 164.0, 163.1, 160.0, 158.0, 152.9, 130.2, 123.9, 123.0, 114.3, 106.8, 98.7, 93.2, 68.3, 55.5, 28.4. ESI-MS *m*/*z* 391.38[M + H]^+^.

#### 7-(2-Bromoethoxy)-3-(4-(2-bromoethoxy)phenyl)-4H-chromen-4-one (a5)

The procedure for the preparation of **a5** was the same with that for **a1–a4**, except that the formononetin was replaced with daidzein and the final product was purified by silico column and eluted by CH_2_Cl_2_. The compound obtained as white solid in 26.3% yield. mp 170.2–172.4 °C; ^1^H NMR (400 MHz, CDCl_3_) *δ* 8.23 (d, *J* = 9.2 Hz, 1H), 7.93 (s, 1H), 7.50 (d, *J* =8.4 Hz, 2H), 7.03–6.97 (m, 3H), 6.87 (d, *J* = 1.6 Hz, 1H), 4.40 (t, *J* = 6.4 Hz, 2H), 4.33 (d, *J* =6.4 Hz, 2H), 3.69 (t, *J* = 6.4 Hz, 2H), 3.66 (t, *J* = 6.4 Hz, 2H); ^13^C NMR (100 MHz, CDCl_3_) *δ*: 174.6, 162.2, 157.7, 157.3, 153.6, 130.1, 127.1, 124.6, 123.3, 117.9, 115.0, 114.4, 101.4, 68.5, 67.8, 31.4, 31.0. ESI-MS (*m*/*z*): 467.44 [M + H]^+^.

#### General procedure for the synthesis of compounds 1–15

Intermediate **a1** (1.87 g, 5 mmol) or **a2** (1.88 g, 5 mmol) or **a3** (2.02 g, 5 mmol) or **a4** (100 mg, 0.26 mmol) were dissolved in 150 ml acetonitrile or 70 ml DMF. Then K_2_CO_3_ (13.8 g, 100 mmol) and various substituted amino-group (16.4 mmol) were added. The mixture was stirred and refluxed for 3 h and monitored by TLC. When the reaction was finished, the reaction solution was poured into 600 ml water and stirred for 5 min. After that, the mixture was filtrated by suction and the pastry was washed by water, then, dried by vacuum at 50 °C to yield the desired products with chromatography, recrystallisation, or without any further purification.

#### 5-Hydroxy-3-(4-mehtoxyphenyl)-7-(2-(piperidin-1-yl)ethoxy)-4H-chromen-4-one (1)

The compound was obtained as white solid in 85.7% yield. Purity 99.3% (by HPLC); mp 81.3–83.1 °C. ^1^H NMR (400 MHz, CDCl_3_) *δ*: 12.83 (s, 1H), 7.86 (s, 1H), 7.46 (d, *J* = 8.4 Hz, 2H), 6.98 (d, *J* = 8.4 Hz, 2H), 6.40 (d, *J* = 13.2 Hz, 2H), 4.20 (brs, 2H), 3.84 (s, 3H), 2.83 (brs, 2H), 2.56 (brs, 4H), 1.65 (brs, 4H), 1.47 (brs, 2H); ^13^C NMR (100 MHz, CDCl_3_) *δ* 181.0, 164.9, 162.8, 159.9, 158.1, 152.8, 130.2, 123.8, 123.1, 114.2, 106.4, 983.9, 93.1, 66.8, 57.7, 55.5, 55.2, 26.0, 24.2. HRMS: calcd for C_23_H_26_NO_5_[M + H]^+^, 396.1811, found: 396.1807.

#### 3-(4-Methoxyphenyl)-7-(3-(piperidin-1-yl)propoxy)-4H-chromen-4-one (2)

The compound was obtained as white solid in 7% yield. Purity 98.8% (by HPLC); mp 152.8–154.0 °C; ^1^H NMR (400 MHz, CDCl_3_) *δ* 8.19 (d, *J* = 8.8 Hz, 1H), 7.91 (s, 1H), 7.50 (d, *J* = 8.8 Hz, 2H), 6.99–6.96 (m, 3H), 6.86 (d, *J* = 2.0 Hz, 1H), 4.12 (t, *J* = 6.4 Hz, 2H), 3.84 (s, 3H), 2.50 (t, *J* = 7.2 Hz, 2H), 2.42 (brs, 4H), 2.07–2.00 (m, 2H), 1.64–1.58 (m, 4H), 1.48–1.44 (m, 2H); ^13^C NMR (100 MHz, CDCl_3_) *δ* 175.9, 163.5, 159.6, 158.0, 152.0, 130.1, 127.7, 124.8, 124.3, 118.3, 114.9, 114.0, 100.6, 67.2, 55.7, 55.3, 54.7, 26.6, 26.0, 24.4. HRMS: calcd for C_24_H_28_NO_4_[M + H]^+^, 394.2018, found: 394.2017.

#### 3-(4-Methoxyphenyl)-7-(4-(piperidin-1-yl)butoxy)-4-H-chromen-4-one (3)

The compound was obtained as white solid in 93.6% yield. Purity 99.4% (by HPLC); mp 152.8–154.0 °C; ^1^H NMR (400 MHz, CDCl_3_) *δ* 8.19 (d, *J* = 8.8 Hz, 1H), 7.91 (s, 1H), 7.51 (d, *J* = 8.8 Hz, 2H), 6.98–6.96 (m, 3H), 6.83 (d, *J* = 2.0 Hz, 1H), 4.08 (t, *J* = 6.4 Hz, 2H), 3.84 (s, 3H), 2.44 (brs, 6H), 1.90–1.83 (m, 2H), 1.78–1.73 (m, 2H), 1.65–1.63 (m, 4H), 1.46 (brs, 2H); ^13^C NMR (100 MHz, CDCl_3_) *δ* 175.9, 163.4, 159.6, 158.0, 152.0, 130.1, 127.8, 124.9, 124.3, 118.3, 114.8, 114.0, 100.6, 68.4, 58.8, 55.4, 54.5, 27.1, 25.7, 24.2, 23.1. HRMS: calcd for C_25_H_30_NO_4_[M + H]^+^, 408.2175, found: 408.21738.

#### 7-(2-(Ethyl(methyl)amino)ethoxy)-3-(4-methoxyphenyl)-4H-chromen-4-one (4)

The compound was obtained as pale yellow solid in 40% yield. Purity 99.2% (by HPLC); mp 110.3–112.4 °C; ^1^H NMR (400 MHz, CDCl_3_) *δ* 8.20 (d, *J* = 8.8 Hz, 1H), 7.91 (s, 1H), 7.49 (d, *J* = 8.8 Hz, 2H), 7.01–6.95 (m, 3H), 6.86 (d, *J* = 2.0 Hz, 1H), 4.18 (t, *J* = 6.0 Hz, 2H), 3.84 (s, 3H), 2.87 (t, *J* = 6.0 Hz, 2H), 2.58 (q, *J* = 7.2 Hz, 2H), 2.38 (s, 3H), 1.12 (t, *J* = 7.2 Hz, 3H); ^13^C NMR (100 MHz, CDCl_3_) *δ* 176.0, 163.3, 159.7, 158.0, 152.2, 130.3, 127.9, 125.0, 124.4, 118.6, 115.0, 114.1, 100.9, 67.0, 55.6, 55.5, 52.1, 42.4, 12.3. HRMS: calcd for C_21_H_24_NO_4_ [M + H]^+^, 354.1705, found: 354.1701.

#### 7-(3-(Ethyl(methyl)amino)propoxy)-3-(4-methoxyphenyl)-4H-chromen-4-one (5)

The compound was obtained as white solid in 26.4% yield. Purity 98.7% (by HPLC); mp 100.7–101.4 °C; ^1^H NMR (400 MHz, CDCl_3_) *δ* 8.19 (d, *J* = 8.8 Hz, 1H), 7.91 (s, 1H), 7.50 (d, *J* = 8.8 Hz, 2H), 6.99–6.96 (m, 3H), 6.86 (d, *J* = 2.0 Hz, 1H), 4.12 (t, *J* = 6.4 Hz, 2H), 3.84 (s, 3H), 2.56 (t, *J* = 6.8 Hz, 2H), 2.47 (q, *J* = 7.2 Hz, 2H), 2.27 (s, 3H), 2.05–1.99 (m, 2H), 1.08(t, *J* = 7.2 Hz, 3H); ^13^C NMR (100 MHz, CDCl_3_) *δ* 176.0, 163.6, 159.7, 158.1, 152.2, 130.3, 127.9, 125.0, 124.4, 118.5, 115.0, 114.1, 100.8, 67.1, 55.5, 53.7, 51.6, 41.8, 27.1, 12.3. HRMS: calcd for C_22_H_26_NO_4_[M + H]^+^, 368.1862, found: 368.1859.

#### 7-(4-(Ethyl(methyl)amino)butoxy)-3-(4-methoxyphenyl)-4H-chromen-4-one (6)

The final compound was obtained as white solid in 11.7% yield purified by medium-pressure preparative column chromatography method (CH_2_Cl_2_: CH3OH =97: 3). Purity 99.1% (by HPLC); mp 128.3–139.9 °C; ^1^H-NMR (400 MHz, CDCl_3_) *δ* 8.17 (d, *J* = 8.8 Hz, 1H), 7.88 (s, 1H), 7.48 (d, *J* = 8.8 Hz, 2H), 6.96–6.94 (m, 3H), 6.81 (d, *J* = 2.0 Hz, 1H), 4.05 (t, *J* = 6.0 Hz, 2H), 3.81 (s, 3H), 2.48–2.41 (m, 4H), 2.24 (s, 3H), 1.88–1.81 (m, 2H), 1.71–1.64 (m, 2H), 1.07 (t, *J* = 7.2 Hz, 3H); ^13^C NMR (100 MHz, CDCl_3_) *δ* 175.9, 163.5, 159.6, 158.0, 152.1, 130.2, 127.7, 124.9, 124.3, 118.3, 114.9, 114.0, 100.6, 68.5, 56.8, 55.4, 51.5, 41.5, 27.0, 23.8, 12.2. HRMS: calcd for C_23_H_28_NO_4_ [M + H]^+^, 382.2018, found: 382.2018.

#### 7-(2-((2-(5-Methoxy-1H-indol-3-yl)ethyl)amino)ethoxy)-3-(4-methoxyphenyl)-4H-chr-omen-4-one (7)

The compound was obtained as pale yellow solid in 10.5% yield. Purity 99.6% (by HPLC); mp 236.0 –238.3 °C; ^1^H NMR (400 MHz, CD_3_OD) *δ* 8.14 (d, *J* = 0.8 Hz, 1H), 8.03 (d, *J* = 8.8 Hz, 1H), 7.46 (d, *J* = 8.8 Hz, 2H), 7.20 (d, *J* = 8.8 Hz, 1H), 7.05 (s, 1H), 7.01 (d, *J* = 2.4 Hz, 1H), 6.96 (d, *J* = 8.8 Hz, 2H), 6.89–6.85 (m, 2H), 6.72 (dd, *J* = 8.8, 2.4 Hz, 1H), 4.13 (t, *J* = 4.2 Hz, 2H), 3.81 (s, 3H), 3.75 (s, 3H), 3.03 (t, *J* = 4.2 Hz, 2H), 2.97 (t, *J* = 3.6 Hz, 4H); ^13^C NMR (100 MHz, CD_3_OD) *δ* 177.9, 164.8, 161.1, 159.5, 154.9, 154.9, 133.5, 131.3, 128.9, 128.2, 125.9, 125.4, 124.4, 119.2, 116.2, 114.8, 113.0, 112.8, 112.6, 101.9, 101.2, 68.3, 56.2, 55.7, 50.3, 48.7, 26.0. HRMS: calcd for C_29_H_29_N_2_O_5_ [M + H]^+^, 485.2076, found: 485.2077.

#### 7-(3-((2-(5-Methoxy-1H-indol-3-yl)ethyl)amino)propoxy)-3-(4-methoxyphenyl)-4H-chromen-4-one (8)

The compound was obtained as yellow solid in 51.8% yield. Purity 98.7% (by HPLC); mp 108.8–109.5 °C; ^1^H NMR (400 MHz, CD_3_OD) *δ* 8.17 (d, *J* = 0.8 Hz, 1H), 8.04 (d, *J* = 8.8 Hz, 1H), 7.47 (d, *J* = 8.4 Hz, 2H), 7.21 (d, *J* = 8.4 Hz, 1H), 7.06–7.05 (m, 2H), 6.98 (d, *J* = 8.8 Hz, 2H), 6.90 (d, *J* = 2.0 Hz, 1H), 6.82 (dd, *J* = 8.8, 2.0 Hz, 1H), 6.75 (dd, *J* = 8.8, 2.0 Hz, 1H), 4.08 (t, *J* = 6.0 Hz, 2H), 3.82 (s, 3H), 3.80 (s, 3H), 2.98 (s, 4H), 2.87 (t, *J* = 6.8 Hz, 2H), 2.05–1.99 (m, 2H); ^13^C NMR (100 MHz, CD_3_OD) *δ* 178.0, 165.0, 161.2, 159.6, 155.0, 154.9, 133.5, 131.4, 128.9, 128.2, 125.9, 125.4, 124.4, 119.1, 116.4, 114.9, 113.1, 112.7, 112.7, 101.7, 101.3, 68.4, 56.3, 55.7, 50.7, 47.5, 29.3, 25.8. HRMS: calcd for C_30_H_31_N_2_O_5_ [M + H]^+^, 499.2233, found: 499.2231.

#### 7-(4((2-(5-Methoxy-1H-indol-3-yl)ethyl)amino)butoxy)-3-(4-methoxyphenyl)-4H-chromen-4-one (9)

The compound was purified by silica column (CH_2_Cl_2_: CH_3_OH= 400: 9) and obtained as yellow solid in 26.1% yield. Purity 99.2% (by HPLC); mp 76.8–79.5 °C; ^1^H NMR (400 MHz, CD_3_OD) *δ* 8.14 (s, 1H), 8.06 (d, *J* = 6.8 Hz, 1H), 7.45 (d, *J* = 8.4 Hz, 2H), 7.21 (d, *J* = 8.8 Hz, 1H), 7.04 (s, 2H), 6.97–6.95 (m, 3H), 6.75 (dd, *J* = 8.8, 2.4 Hz, 1H), 4.03 (t, *J* = 6.0 Hz, 2H), 3.80 (s, 3H), 3.80 (s, 3H), 2.98 (brs, 4H), 2.70 (t, *J* = 7.2 Hz, 2H), 1.82–1.75 (m, 2H), 1.71–1.64 (m, 2H); ^13^C NMR (100 MHz, CD_3_OD) *δ* 178.0, 165.3, 161.2, 159.7, 155.0, 154.9, 133.5, 131.4, 128.9, 128.2, 125.9, 125.4, 124.3, 119.0, 116.4, 114.9, 113.0, 113.0, 112.7, 101.8, 101.3, 69.6, 56.3, 55.7, 50.7, 49.9, 27.7, 26.8, 25.9. HRMS calcd for C_31_H_33_N_2_O_5_ [M + H]^+^, 513.2389, found: 513.2380.

#### 7-(2-(Benzyl(methyl)amino)ethoxy)-3-(4-methoxyphenyl)-4H-chromen-4-one (10)

The compound was obtained as white solid in 76.1% yield. Purity 98.8% (by HPLC); mp 135.0–136.6 °C; ^1^H NMR (400 MHz, DMSO-d_6_) *δ*: 8.42 (s, 1H), 8.02 (d, *J* = 8.8 Hz, 1H), 7.53 (d, *J* = 8.4 Hz, 2H), 7.32–7.24 (m, 5H), 7.18 (s, 1H), 7.08 (d, *J* = 8.8 Hz, 1H), 7.00 (d, *J* = 8.4 Hz, 2H), 4.26 (t, *J* = 5.6 Hz, 2H), 3.79 (s, 3H), 3.59 (s, 2H), 2.79 (t, *J* = 5.6 Hz, 2H), 2.25(s, 3H); ^13^C NMR (100 MHz, DMSO-d_6_) *δ* 174.6, 162.9, 159.0, 157.4, 153.5, 138.9, 130.1, 128.1, 126.9, 126.9, 124.1, 123.4, 117.6, 115.1, 113.6, 101.1, 66.8, 61.6, 55.1, 54.9, 42.3. HRMS: calcd for C_26_H_26_NO_4_[M + H]^+^, 416.1862, found: 416.1855.

#### 7-(2-(Dibenzylamino)ethoxy)-3-(4-methoxyphenyl)-4H-chromen-4-one (11)

The crude product was recrystallised by ethyl acetate. The final product was obtained as white solid in 49.9% yield. Purity 98.4% (by HPLC); mp 118.9–120.0 °C; ^1^H NMR (400 MHz, DMSO-d_6_) *δ*: 8.42 (s, 1H), 8.02 (d, *J* = 8.8 Hz, 1H), 7.53 (d, *J* = 8.4 Hz, 2H), 7.39 (d, *J* = 7.6 Hz, 4H), 7.32 (d, *J* = 7.6 Hz, 4H), 7.23 (t, *J* = 7.2 Hz, 2H), 7.09 (s, 1H), 7.05–6.99 (m, 3H), 4.25 (t, *J* = 5.6 Hz, 2H), 3.79 (s, 3H), 3.68 (s, 4H), 2.83 (t, *J* = 5.6 Hz, 2H); ^13^C NMR (100 MHz, DMSO-d_6_) *δ* 174.6, 162.8, 159.0, 157.4, 153.4, 139.2, 130.0, 128.5, 128.2, 126.9, 126.9, 124.1, 123.3, 117.5, 115.1, 113.6, 101.0, 66.7, 57.9, 55.1, 51.2. HRMS: calcd for C_32_H_30_NO_4_ [M + H]^+^, 492.2175, found: 492.2169.

#### 3-(4-Methoxyphenyl)-7-(2-(prop-2-yn-1-ylamino)ethoxy)-4H-chromen-4-one (12)

The compound was obtained as yellow solid in 54.1% yield. Purity 99.4% (by HPLC); mp 127.8–130.7 °C; ^1^H NMR (400 MHz, CD_3_COCD_3_) *δ* 8.22 (s, 1H), 8.11 (d, *J* = 8.4 Hz, 1H), 7.57 (d, *J* = 7.6 Hz, 2H), 7.09–6.97 (m, 4H), 4.28 (t, *J* = 4.8 Hz, 2H), 3.31 (s, 3H), 3.49 (s, 2H), 3.12 (t, *J* = 4.8 Hz, 2H), 2.66 (s, 1H); ^13^C NMR (100 MHz, CD_3_COCD_3_) *δ* 175.6, 164.4, 160.5, 158.8, 153.6, 131.0, 128.1, 125.4, 125.1, 119.2, 115.7, 114.4, 101.7, 83.2, 72.7, 69.4, 55.6, 47.7, 38.5. HRMS: calcd for C_21_H_30_NO_4_[M + H]^+^, 350.1392, found: 350.1383.

#### 7-(2-(((3 R,5 S,7r)-3,5-dimethyladamantan-1-yl)amino)ethoxy)-3-(4-methoxyphenyl)-4H-chromen-4-one (13)

The compound was obtained as pale yellow solid in 41% yield. Purity 98.9% (by HPLC); mp 127.5–129.5 °C; ^1^H NMR (400 MHz, CDCl_3_) *δ* 8.20 (d, *J* = 8.8 Hz, 1H), 7.91 (s, 1H), 7.49 (d, *J* = 8.8 Hz, 2H), 7.00–6.96 (m, 3H), 6.84 (d, *J* = 1.6 Hz, 1H), 4.15 (t, *J* = 5.6 Hz, 2H), 3.84 (s, 3H), 3.04 (t, *J* = 5.6 Hz, 2H), 2.18–2.15 (m, 1H), 1.52 (d, *J* = 2.0 Hz, 2H), 1.35–1.27 (m, 9H), 2.21 (dd, *J* = 12.4, 7.2 Hz, 2H), 0.86 (s, 6H); ^13^C NMR (100 MHz, CDCl_3_) *δ* 176.0, 163.4, 159.7, 158.0, 152.2, 130.3, 127.9, 125.0, 124.4, 118.6, 115.0, 114.1, 100.8, 69.5, 55.5, 52.4, 51.1, 49.2, 43.1, 41.4, 39.8, 32.6, 30.5, 30.4. HRMS calcd for C_30_H_36_NO_4_ [M + H]^+^, 474.2644, found: 474.2638.

#### 3-(4-Hydroxyphenyl)-7-(2-(piperidin-1-yl)ethoxy)-4H-chromen-4-one^.^HBr (14)

The product from the prior step (569 mg, 1.5 mmol) was dissolved into 10 ml 40% methanol solution of HBr and refluxed at 80 °C for 3 h. After cooling down to 0 °C, the reactant was filtrated by suction and washed by methanol (3 ml) for three times. Then, the liquid was removed by vacuum and the residues were recrystallised by methanol. The compound was obtained as white solid in 15.8% yield. Purity 99.3% (by HPLC); mp 241.2–246.5 °C; ^1^H NMR (400 MHz, DMSO-d_6_) *δ* 8.39 (s, 1H), 8.04 (d, *J* = 8.8 Hz, 1H), 7.40 (d, *J* = 8.0 Hz, 2H), 7.21 (s, 1H), 7.10 (d, *J* = 8.8 Hz, 1H), 6.82 (d, *J* = 8.0 Hz, 2H), 4.34 (t, *J* = 4.2 Hz, 2H), 3.03 (brs, 2H), 2.77 (brs, 4H), 1.64–1.58 (m, 4H), 1.44 (brs, 2H); ^13^C NMR (100 MHz, D_2_O/CD_3_OD) *δ* 178.7, 163.4, 159.2, 157.4, 155.5, 131.5, 128.2, 125.6, 124.2, 119.1, 116.4, 116.4, 102.4, 63.3, 56.4, 54.7, 23.7, 22.1. HRMS: calcd for C_22_H_24_NO_4_ [M − Br]^+^, 366.1705, found: 366.1694.

#### 3-(4-Hyroxyphenyl)-7-(2-(pyrrolidin-1-yl)ethoxy)-4H-chromen-4-one ^.^HBr (15)

The procedure for the synthesis of compound **15** was the same as that of **14**. The compound was obtained as white solid in 15.8% yield. Purity 98.9% (by HPLC); mp 234.4–236.8 °C; ^1^H NMR (400 MHz, D_2_O/CD_3_OD) *δ* 8.15(s, 1H), 8.01 (d, *J* = 8.8 Hz, 1H), 7.36 (d, *J* = 8.4 Hz, 2H), 7.12 (d, *J* = 8.8 Hz, 1H), 7.07 (s, 1H), 6.93 (d, *J* = 8.4 Hz, 2H), 4.38 (t, *J* = 4.4 Hz, 2H), 3.62 (t, *J* = 4.4 Hz, 2H), 3.45 (brs, 4H), 2.14 (brs, 4H); ^13^C NMR (100 MHz, D_2_O/CD_3_OD) *δ* 178.4, 163.4, 159.0, 157.4, 155.3, 131.4, 128.2, 125.4, 124.1, 119.0, 116.3, 116.3, 102.3, 64.6, 55.6, 54.5, 23.7. HRMS: calcd for C_21_H_22_NO_4_ [M − Br]^+^, 352.1543, found: 352.1541.

#### 7-(2-(Piperidin-1-yl)ethoxy)-3-(4-(2-(piperidin-1-yl)ethoxy)phenyl)-4H-chrom en-4-one (16)

Intermediate **a5** (932 mg, 2 mmol) was dissolved in 50 ml acetonitrile. Then K_2_CO_3_ (4.60 g, 33.2 mmol) and piperidine (0.31 ml, 3.13 mmol) were added. The mixture was refluxed and stirred overnight. When the reaction ended, the reaction mixture was poured into 200 ml water and stirred for 30 min. After that, the mixture was filtrated by suction and the pastry was washed by water (6 ml) for three times. The crude product was purified by 200–300 mush silica column (CH_2_Cl_2_:CH_3_OH =20:3). The purified compound was obtained as white solid in 13.6% yield. Purity 99.6% (by HPLC); mp 148.7–152.2 °C; ^1^H NMR (400 MHz, CDCl_3_) *δ* 8.19(d, *J* = 9.2 Hz, 1H), 7.91 (s, 1H), 7.48 (d, *J* = 8.8 Hz, 2H), 7.00–6.96 (m, 3H), 6.86 (d, *J* = 2.4 Hz, 1H), 4.20 (t, *J* = 6.0 Hz, 2H), 4.15 (t, *J* = 6.0 Hz, 2H), 2.84–2.79 (m, 4H), 2.53 (brs, 8H), 1.65–1.59 (m, 8H), 1.46 (brs, 4H); ^13^C NMR (100 MHz, CDCl_3_) *δ* 176.0, 163.3, 158.9, 158.0, 152.2, 130.2, 127.9, 125.0, 124.4, 118.6, 115.1, 114.8, 100.9, 66.9, 66.1, 58.0, 57.8, 55.3, 55.2, 26.1, 26.0, 24.3, 24.3. HR MS: calcd for C_29_H_37_N_2_O_4_ [M + H]^+^, 477.2753, found: 477.2743.

#### 3-(4-Methoxyphenyl)-7-(2-oxo-2-(piperidin-1-yl)ethoxy)-4H-chromen4-one (17)

The piperidine (4 ml, 80 mmol) which was diluted in THF (5 ml) was added dropwise into the chloroacetyl chloride (3.1 ml, 40 mmol) dissolved in THF (10 ml) and stirred for 10 h at room temperature, the mixture was condensed by reduced pressure to dryness and the residue was dissolved in 50 ml acetone. Then, formononetin (1 g, 3.73 mmol) and K_2_CO_3_ (4.60 g, 33.2 mmol) were added into the solution. The reactant was refluxed overnight. When the reaction finished, the reaction solution was poured into 200 ml water, stirred and filtrated by suction. The pastry was washed by water and purified by 200–300 mush silica column (CH_2_Cl_2_:CH_3_OH =100:1) .The compound was obtained as pale yellow solid in 45.3% yield. Purity 99.0% (by HPLC); mp 144.8–145.8 °C; ^1^H NMR (400 MHz, CDCl_3_) *δ* 8.16 (d, *J* = 8.8 Hz, 1H), 7.87 (s, 1H), 7.45 (d, *J* = 8.8 Hz, 2H), 6.99 (d, *J* = 8.8 Hz, 1H), 6.93–6.89 (m, 3H), 4.75 (s, 2H), 3.79 (s, 3H), 3.53 (brs, 2H), 3.42 (brs, 2H), 1.62–1.51 (m, 6H); ^13^C NMR (100 MHz, CDCl_3_) *δ* 175.7, 165.0, 162.3, 159.5, 157.7, 152.2, 130.1, 127.9, 124.8, 124.1, 118.9, 114.6, 113.9, 101.4, 67.5, 55.3, 46.3, 43.2, 26.5, 25.5, 24.3. HRMS calcd for C_25_H_30_NO_5_ [M + H]^+^, 424.2124, found: 424.2124.

#### 3-(4-Methoxyphenyl)-7-(3-oxo-3-(piperidin-1-yl)propoxy)-4H-chromen-4-one (18)

K_2_CO_3_ (7.5 g, 54.3 mmol), 3-bromopropionic acid (6 g, 39.2 mmol) and formononetin (2 g, 7.4 mmol) were dissolved in 100 ml acetone and refluxed for 2 days. After that, the reactant was condensed by reduced pressure to dryness and then the residue was poured into 400 ml water. In order to precipitate the product, the solution should be acidified with HCl (1 N) to pH 5. Then, the precipitated white solid was filtrated by suction and washed with water (5 ml) for three times. 3-((3-(4-Methoxyphenyl)-4H-chromogen 4-one-7-yl)oxy)propanoic acid crude product (2.465 g) was obtained.

The obtained crude product was dissolved in 50 ml DMF, then, N,N-diisopropylethylamine (1.02 ml, 5.85 mmol), piperidine (0.5 ml, 5.1 mmol) and hydrochloro-1-(3-dimethylaminopropyl)-3-ethylcarbodiimide salt were added and stirred overnight at room temperature. When the reaction finished, the reactant was poured into 200 ml water and extracted with CH_2_Cl_2_, washed with saturated K_2_CO_3_ and brine respectively. The organic phase was taken, dried with Na_2_SO_4_, filtrated by suction and the filtrate was condensed by reduced pressure. The residues were purified by silica column (petroleum ether:ethyl acetate =2:1). The final compound was obtained as pale yellow solid in 26.9% yield. Purity 99.5% (by HPLC); mp 161.0–163.0 °C; ^1^H NMR (400 MHz, CDCl_3_) *δ* 8.19 (d, *J* = 8.8 Hz, 1H), 7.91 (s, 1H), 7.50 (d, *J* = 8.4 Hz, 2H), 6.96 (brd, *J* = 8.4 Hz, 3H), 6.90 (s, 1H), 4.42 (t, *J* = 6.0 Hz, 2H), 3.84 (s, 3H), 3.54 (brd, 4H), 2.89 (t, *J* = 6.0 Hz, 2H), 1.70–1.60 (m, 6H); ^13^C NMR (100 MHz, DMSO-d_6_) *δ* 174.6, 167.6, 162.9, 159.0, 157.4, 153.5, 130.1, 126.9, 124.1, 123.3, 117.5, 115.0, 113.6, 101.0, 65.1, 55.1, 45.9, 42.0, 31.8, 26.0, 25.3, 24.0. HRMS: calcd for C_24_H_26_NO_5_ [M + H]^+^, 408.1811, found: 408.1805.

#### 2-((3-(4-Methoxyphenyl)-4-oxo-4H-chromen-7-yl)oxy)ethyl piperidine-1-carboxylate (19)

Intermediate **a1** (0.748 mg, 2 mmol), K_2_CO_3_ (4.60 g, 33.3 mmol) was dissolved in DMF (70 ml), then, piperidine (0.302 ml, 3.30 mmol) was poured into the reactant liquid, stirred and refluxed for 3 h. When the reaction finished, the mixture was poured into water (300 ml), stirred for 30 min and filtrated by suction. The pastry was washed by water (5 ml) for three times and purified by 200–300 mush silica column eluted by CH_2_Cl_2_. The compound was obtained as pale yellow solid in 12.6% yield. Purity 99.1% (by HPLC); mp 104.5–109.4 °C. ^1^H NMR (400 MHz, CDCl_3_) *δ* 8.19 (d, *J* = 8.8 Hz, 1H), 7.90 (s, 1H), 7.48 (d, *J* = 8.8 Hz, 2H), 7.00 (dd, *J* = 8.8, 2.0 Hz, 1H), 6.95 (d, *J* = 8.8 Hz, 2H), 6.86 (d, *J* = 2.0 Hz, 1H), 4.46 (t, *J* = 6.0 Hz, 2H), 4.27 (t, *J* = 6.0 Hz, 2H), 3.82 (s, 3H), 3.41 (brs, 4H), 1.56–1.51 (m, 6H); ^13^C NMR (100 MHz, CDCl_3_) *δ* 175.1, 163.0, 159.5, 157.8, 155.0, 152.1, 130.1, 127.8, 124.8, 124.2, 118.6, 114.8, 113.9, 100.9, 67.0, 63.1, 55.3, 44.9, 25.6, 24.3. HRMS: calcd for C_24_H_26_NO_6_ [M + H]^+^, 424.1760, found: 424.1751.

#### 2-((3-(4-Methoxyphenyl)-4-oxo-4H-chromen-7-yl)oxy)ethyl prop-2-yn-1-ylc-arbamate (20)

The procedure was the same as above. However, the piperidine was replaced with prop-2-yn-1-amine (0.226 ml, 3.30 mmol). The final compound was obtained as pale yellow solid in 18.7% yield. Purity 99.4% (by HPLC); mp 155.1–157.4 °C; ^1^H NMR (400 MHz, DMSO-d_6_) *δ* 8.42 (s 1H), 8.03 (d, *J* = 8.8 Hz, 1H), 7.78 (t, *J* = 5.6 Hz, 1H), 7.53 (d, *J* = 8.8, 2.0 Hz, 2H), 7.20 (d, *J* = 2.0 Hz, 1H), 7.10 (dd, *J* = 8.8, 2.0 Hz, 1H), 7.01–6.97 (m, 2H), 4.37–4.32 (m, 4H), 3.79 (s, 5H), 3.10 (t, *J* = 2.4 Hz, 1H); ^13^C NMR (100 MHz, DMSO-d_6_) *δ* 174.6, 162.6, 159.0, 157.4, 155.8, 153.5, 130.1, 127.0, 124.0, 123.4, 117.7, 115.0, 113.6, 101.2, 81.3, 73.0, 67.1, 62.5, 55.1, 29.8. HRMS: calcd for C_22_H_20_NO_6_ [M + H]^+^, 394.1291, found: 394.1286.

### AChE/BuChE bioassay

To measure *in vitro* AChE/BuChE activity, modified Ellman's method^27–30^ was performed using a 96-well plate reader (BioTek ELx808). AChE (0.5 U/mg) was extracted from rat cortex while BuChE (3.4 U/mg) was obtained from human plasma. Each well contained 50 μl potassium phosphate buffer (KH_2_PO_4_/K_2_HPO_4_, 0.1 M, pH 8.0), 25 μl test compounds and 25 μl enzyme. Notably, the test compounds were firstly dissolved in the mixture of 50% methanol and 50% DMSO, and eventually diluted so that the final concentration of both solvents was less than 1% in the assay. They were pre-incubated for 60 min at 37 °C, and then 125 μl 5,5′-dithiobis-2-nitrobenzoic acid (DNTB, 3 mM in buffer) was added. Characterisation of the hydrolysis of acetylthiocholine iodide or butyrylthiocholine chloride catalysed by AChE/BuChE was performed spectrometrically at 412 nm, followed by the addition of substrate (acetylthiocholine iodide or butyrylthiocholine chloride 3 mM in water, respectively). The activity was determined by measuring the increase in absorbance at 412 nm after 15 min. For those compounds with inhibition rate >50% at the concentration of 50 μM, the IC_50_ values were further determined. A control experiment was performed under the same condition without any inhibitor and the blank contained buffer, water, DTNB and substrate. The described method was also performed for BuChE bioassay. In the bioassay, donepezil (an AChE inhibitor) and tetraisopropyl pyrophosphoramide (Iso-OMPA, a BuChE inhibitor) were used as positive controls of AChE and BuChE, respectively.

### Molecular docking

In order to understand the protein-ligand interactions between the synthesised isoflavone derivatives and AChE/BuChE, the most potent inhibitor as an example was docked against AChE and BuChE. The ligand structure was built and prepared by DS 2016 (Discovery Studio version 2016, San Diego, CA, USA). Hydrogen atoms were added to the structure and its ionisation states at pH 7.3 to 7.5 were generated. Besides, its lowest-energy conformation was generated prior to docking. The X-ray structures of AChE (PDB ID: 5EIH)^31^ and BuChE (PDB ID: 4BDS)^32^ were retrieved from the Protein Data Bank. Both of them were high-resolution structures of protein-ligand complexes and their organisms were consistent with those of the enzymes used for bioassay. After stripping the cognate ligand from each complex, molecular docking was carried out by GOLD 3.0.1 in which the target protein was kept rigid, while the ligands were left flexible to explore the conformational space inside the binding site. After that, the docking poses of the ligand were visually inspected.

### In silico prediction of pharmacokinetic properties

Pharmacokinetic properties, in particular blood–brain barrier penetration (BBB), are quite important for the drugs for the treatment of AD. Therefore, the pharmacokinetic properties of AChE/BuChE inhibitors were predicted by the “ADMET Descriptors” module implemented in DS2016. The pharmacokinetic properties available in this module were aqueous solubility, BBB, CYP2D6 binding, hepatotoxicity, intestinal absorption and plasma protein binding.

## Results and discussion

### Chemistry

In total, 20 isoflavone derivatives (cf. Table 1) were synthesised and their chemical structures were confirmed by melting point, HR MS, ^1^H NMR and ^13^C NMR.

**Table 1.** Chemical structures of synthesised isoflavone derivatives.


Compound	*n*	R1	R2	R
**1**	2	OH	CH_3_	
**2**	3	H	CH_3_	
**3**	4	H	CH_3_	
**4**	2	H	CH_3_	
**5**	3	H	CH_3_	
**6**	4	H	CH_3_	
**7**	2	H	CH_3_	
**8**	3	H	CH_3_	
**9**	4	H	CH_3_	
**10**	2	H	CH_3_	
**11**	3	H	CH_3_	
**12**	4	H	CH_3_	
**13**	2	H	CH_3_	
**14**	2	H	H	
**15**	2	H	H	
**16**	2	H		
**17**	1	H	CH_3_	
**18**	2	H	CH_3_	
**19**	2	H	CH_3_	
**20**	2	H	CH_3_	

**Scheme 1.**
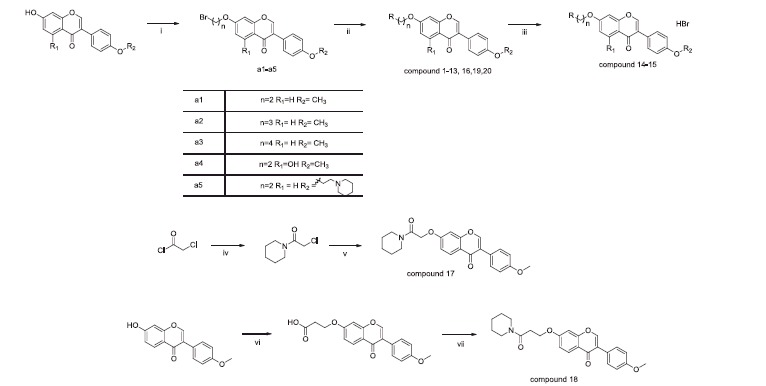
The general procedure for the synthesis of compounds **1**–**20**. (i): K_2_CO_3_, acetone, Br(CH_2_)_n_Br, 60 °C; (ii): RH (amines), K_2_CO_3_, DMF/Acetonitrile, 100 °C, 3 h; (iii): 40% HBr, 120 °C, 3 h; (iv): piperidine, THF r.t. 10 h; (v): formononetin, K_2_CO_3_, acetone; (vi) K_2_CO_3_, 3-bromopropionic acid, acetone, 60 °C, 2 days; (vii) DMF, N, N-diisopropylethylamine, piperidine, 12 h, r.t.

The synthetic route of new isoflavone derivatives was designed and depicted in Scheme 1 according to earlier reports^33^^,^^34^. Briefly, the bromo-substituted intermediates **a1–a5** were prepared through commercially available isoflavones and dibromoalkane in acetone in the presence of potassium carbonate. Reaction between **a1–a5** and corresponding amines in acetonitrile afforded compounds **1**–**16**. At first, we synthesised compounds **1**–**9** and evaluated their biological activity. Structure-activity relationship (SAR) of these compounds demonstrated the optimal length of the linker between the amino group and isoflavone core structure seems to be 2. Previous reports indicated that various amino groups can form a cation–pi interaction with the aromatic amino acid residues (i.e. Trp84 in AChE and Try82 in BuChE) located in the catalysis active site of the cholinesterases^35^. This observation prompted us to design and synthesise compounds **10–20** by substitution of different amino groups.

It is worth noting that carbamate substituted compounds **19** and **20** can be produced by the reaction of amines and bromo-substituted isoflavone in the presence of DMF and K_2_CO_3_ rather than the reported reaction of acyl chloride with hydroxyl-substituted isoflavone^36^ in acetonitrile. Interestingly, compound **17** can be synthesised by step 1 and step 2 as shown in Scheme 1, however this synthetic route was not suitable for compound **18** because of the poor reactive activity of 3-chloro-1-(piperidin-1-yl)propan-1-one which has a longer distance between reactive site and amide compared to 2-chloro-1-(piperidin-1-yl) ethan-1-one. Therefore, the intermediate 3-((3-(4-methoxyphenyl)-4H-chromogen-4-one-7-yl)oxy)propanoic acid was obtained by the reaction of formononetin with 3-bromopropionic acid. Piperidine was then added to the intermediate to produce the final compound **18**.

### *In vitro* inhibition of AChE and BuChE

Both *in vitro* AChE and BuChE inhibitory activity were evaluated for all the synthesised isoflavone derivatives. The results are shown in Table 2. To be noted donepezil and Iso-OMPA were used as the positive drugs.

**Table 2.** *In vitro* inhibition of AChE and BuChE for compounds **1**–**20**.

	Inhibition % at 50 μM		IC_50_ (μM)	
Name	AChE	BuChE	AChE	BuChE
**G**	n.d.^a^	n.d.	1.47	3.37
**1**	71.10	95.74	10.3	4.12
**2**	28.34	97.00	n.d.	23.2
**3**	85.65	98.11	46.39	11.1
**4**	58.66	84.13	100.4	141.3
**5**	25.38	91.32	n.d.	103.6
**6**	63.76	94.77	106.6	68.08
**7**	3.68	45.74	n.d.	n.d.
**8**	2.42	60.63	n.d.	15.6
**9**	0.72	37.92	n.d.	n.d.
**10**	3.34	2.72	n.d.	n.d.
**11**	−6.27	−18.48	n.d.	n.d.
**12**	5.47	−16.87	n.d.	n.d.
**13**	17.61	−6.62	n.d.	n.d.
**14**	79.38	99.86	9.75	7.66
**15**	75.22	99.88	57.74	7.19
**16**	100.34	99.74	4.60	5.92
**17**	0.54	−2.83	n.d.	n.d.
**18**	−2.36	5.59	n.d.	n.d.
**19**	−11.56	53.03	n.d.	9.59
**20**	−4.84	−5.49	n.d.	n.d.
Donepezil	64.82	n.d.	1.05	n.d.
Iso-OMPA	n.d.	94.28	n.d.	5.78

a n.d.: not determined.

As shown in Table 2, five isoflavone derivatives, i.e. **1**, **3**, **14**, **15** and **16**, showed both AChE and BuChE inhibitory effect. Notably, compound **16** displayed equivalent inhibitory activity to two single-targeting drugs (Donepezil and Iso-OMPA) as well as our hit compound **G**.

Based on the chemical structures and their biological activity, it is clear that AChE and BuChE inhibitory activities changed markedly because of the change of amino substitutes. Compounds would manifest inhibitory effect when C_7_ was substituted by piperidine or N-methylethanamide. In the meanwhile, the length of the side chain also played a significant role in maintaining the inhibitory activity. Generally, compounds with side chains of two or four carbon atoms can display a dual-targeting inhibition whereas those with three carbon atoms selectively inhibited BuChE. Besides, compounds with a side chain of four carbon atoms showed stronger inhibition for BuChE than those with two carbon atoms. However, ester-substituted compounds displayed no AChE inhibitory effect. The alternative isoflavone core changed the inhibition level for both AChE and BuChE. C_5_ hydroxyl substitution improved the inhibitory effect. When C_4′_ was substituted for 2-piperidineethoxyl group, the most potent AChE inhibitor **16** was obtained. All the information provided us with clues for further lead optimisation.

### Binding modes of isoflavone derivatives

**Figure 3.**
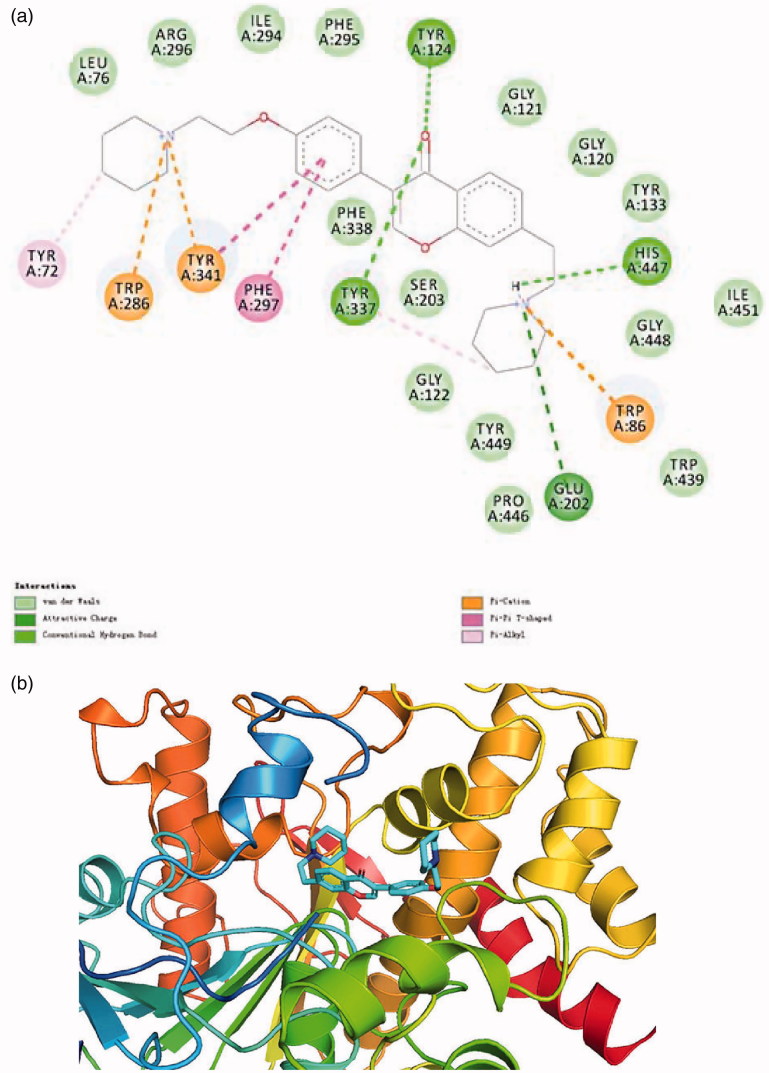
(a) 2D schematic diagram of potential interactions between compound **16** and AChE. (b) The predicted binding mode of compound **16** with AChE.

As compound **16** was the most potent AChE/BuChE dual-targeted inhibitor, it was selected as an example and used in the docking simulation. As shown in Figure 3, compound 16 was able to bind both AChE and BuChE. Regarding AChE, the piperidine moiety in C_4_’ position occupied the “entrance cavity” by forming π–cation interaction with the key amino acid residues TRP286 and TYR341, while the isoflavone core structure was well accommodated in the catalytic cleft through π–π T-shaped interactions with PHE297/TYR341 and hydrogen bonds with TYR124/TYR337. Furthermore, the π–cation interaction between the piperidine ring in C_7_ position with TRP86, the salt bridge with GLU202 and the hydrogen bond with HIS447 also contributed to the stabilisation of protein–ligand interaction.

**Figure 4.**
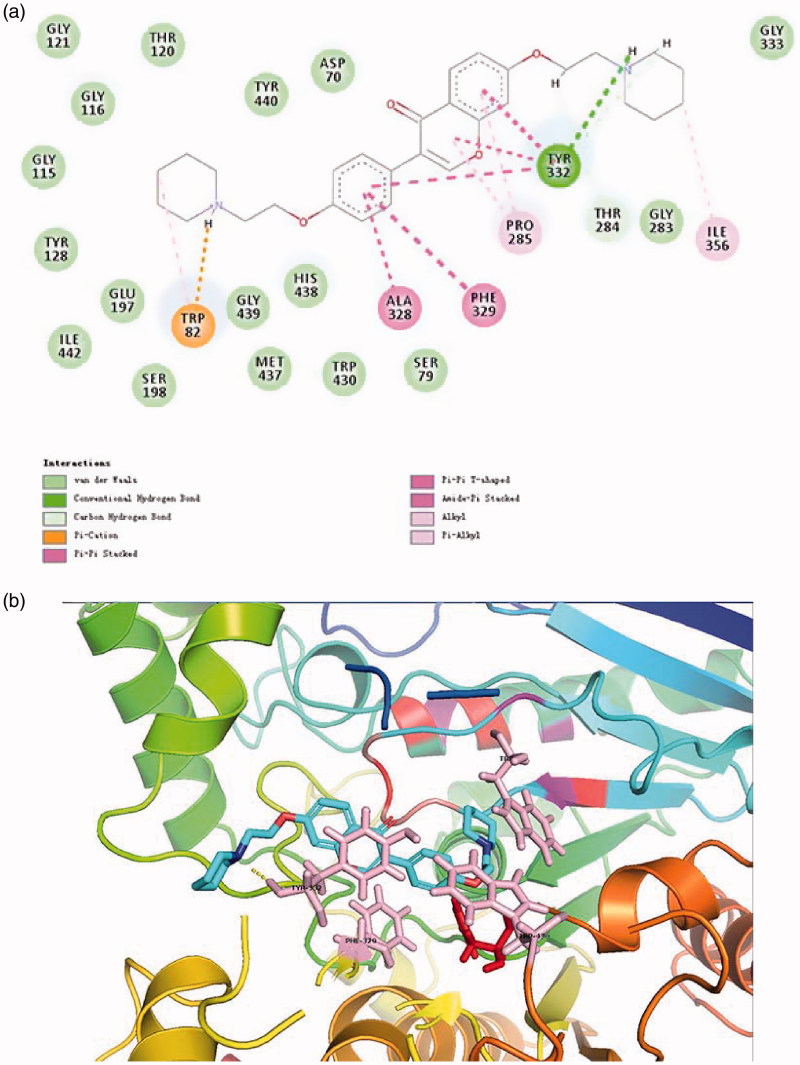
(a) 2D schematic diagram of potential interactions between compound **16** and BuChE. (b) The predicted binding mode of compound **16** with BuChE.

Protein–ligand interactions between compound **16** with BuChE were similar to that for AChE (cf. Figure 4). It deserved to mention that the piperidine moiety in C_7_ position formed a hydrogen bond with the key amino acid residue TYR332 which can also interact with the isoflavone structure through π–π stacking and π–π T-shape. Protonated amino group in C_4_ position can stabilise the complex through forming a salt bridge with both TRP82.

### Predicted pharmacokinetic properties

As shown in Table 3, all the AChE/BuChE dual-targeted inhibitors reported in this study appeared to have poor solubility in aqueous media whereas possess good absorption. Like compound **G**, compounds **3**, **14** and **16** were predicted to penetrate the BBB, which was a favoured property for drugs to treat neurodegenerative diseases, e.g. AD. Encouragingly, compound 16 may not bind to CYP2D6, which was different from other compounds, including the hit compound **G**. This unique feature would be beneficial for ensuring the efficacy of compound **16** and avoiding the side-effect. These data prove our hit-to-lead optimisation strategy seems to be also effective in optimising pharmacokinetic property.

**Table 3.** Predicted pharmacokinetic properties of compounds **1**, **3**, **14**, **15** and **16.**

Compound	AlogP98	PSA-2D	Solubility level	Absorption level	BBB level	PPB	CYP2D6
**1**	3.647	68.259	2	0	2	True	True
**3**	4.532	47.443	2	0	1	True	True
**14**	3.664	59.328	2	0	1	True	True
**15**	3.208	59.328	2	0	2	True	True
**16**	4.945	50.796	2	0	1	True	False
**G**	4.267	47.443	2	0	1	True	True

AlogP98: Lipophilicity descriptor; PSA-2D: Polar surface area; AlogP98: Lipophilicity descriptor; PSA-2D: Polar surface area; Solubility Level: (0, Good; 1, Moderate; 2, Poor; 3, Very poor); Absorption Level: (0, Good; 1, Moderate; 2, Poor; 3, Very poor); BBB Level: (0, very high blood–brain barrier penetration; 1, high; 2, medium; 3, low).

## Conclusion

In this study, we report the synthesis of a series of novel isoflavone derivatives based on our hit compound G identified by *in silico* HTS. The *in vitro* AChE/BuChE bioassay has shown 5 out of 20 isoflavone derivatives were AChE/BuChE dual-targeted inhibitors. SAR analysis has demonstrated the length of the side chain and the type of amino-substituted group played an essential role in AChE inhibition, whereas both factors had little effect on the BuChE inhibition. Among these derivatives, compound **16** possessed the greatest AChE inhibition as well as strong BuChE inhibitory activity. The molecular docking study demonstrated that π–cation and π–π interactions were essential for compound **16** to display its dual-targeting effect. In addition, the *in silico* prediction of pharmacokinetic properties has indicated 1) compounds **3**, **14** and **16** were able to penetrate the BBB and 2) compound **16** may be more effective and less toxic due to no binding to CYP2D6. Taken together, compound **16** may warrant further development as a potential drug-like AChE/BuChE dual-targeted inhibitor for the treatment of AD.

## Supplementary Material

IENZ_1347163_Supplementary_Material.pdf

## References

[1] Kumar A, Singh A. A review on Alzheimer's disease pathophysiology and its management: an update. Pharmacol Rep 2015;67:195–203. 25712639 10.1016/j.pharep.2014.09.004

[2] Anand R, Gill KD, Mahdi AA. Therapeutics of Alzheimer's disease: past, present and future. Neuropharmacology 2014;76 Pt A:27–50. 10.1016/j.neuropharm.2013.07.004 23891641

[3] Geula C, Mesulam MM. Cholinesterases and the pathology of Alzheimer disease. Alzheimer Dis Assoc Disord 1995;9 Suppl 2:23–8. 8534419 10.1097/00002093-199501002-00005

[4] Overk CR, Felder CC, Tu Y, et al Cortical M1 receptor concentration increases without a concomitant change in function in Alzheimer's disease. J Chem Neuroanat 2010;40:63–70. 20347961 10.1016/j.jchemneu.2010.03.005 PMC2864794

[5] Yu Q-S, Holloway HW, Flippen-Anderson JL, et al Methyl analogues of the experimental alzheimer drug phenserine: synthesis and structure/activity relationships for acetyl- and butyrylcholinesterase inhibitory action. J Med Chem 2001;44:4062–71. 11708910 10.1021/jm010080x

[6] Silva T, Reis J, Teixeira J, Borges JF. Alzheimer's disease, enzyme targets and drug discovery struggles: from natural products to drug prototypes. Ageing Res Rev 2014;15:116–45. 24726823 10.1016/j.arr.2014.03.008

[7] Pepeu G, Giovannini MG. Cholinesterase inhibitors and memory. Chem Biol Interact 2010;187:403–8. 19941841 10.1016/j.cbi.2009.11.018

[8] AnandSingh PB. A review on cholinesterase inhibitors for Alzheimer's disease. Arch Pharm Res 2013;36:375–99. 23435942 10.1007/s12272-013-0036-3

[9] Houghton PJ, Ren Y, Howes MJ. Acetylcholinesterase inhibitors from plants and fungi. Nat Prod Rep 2006;23:181–99. 16572227 10.1039/b508966m

[10] Munoz FJ, Inestrosa NC. Neurotoxicity of acetylcholinesterase amyloid beta-peptide aggregates is dependent on the type of abeta peptide and the ache concentration present in the complexes. FEBS Lett. 1999;450:205–9. 10359075 10.1016/s0014-5793(99)00468-8

[11] Sugimoto H Donepezil hydrochloride: a treatment drug for Alzheimer's disease. Chem Rec 2001; 1: 63–73. 11893059 10.1002/1528-0691(2001)1:1<63::AID-TCR9>3.0.CO;2-J

[12] Zarotsky V, Sramek JJ, Cutler NR. Galantamine hydrobromide: an agent for Alzheimer's disease. Am J Health Syst Pharm 2003;60:446–52. 12635450 10.1093/ajhp/60.5.446

[13] CastroMartinez AA. Targeting beta-amyloid pathogenesis through acetylcholinesterase inhibitors. Curr Pharm Des 2006;12:4377–87. 17105433 10.2174/138161206778792985

[14] Birks J. Cholinesterase inhibitors for Alzheimer's disease. Cochrane Database Syst Rev 2006;1:CD005593. 10.1002/14651858.CD005593 PMC9006343 16437532

[15] Darvesh S, Hopkins D, Geula AC. Neurobiology of butyrylcholinesterase. Nat Rev Neurosci 2003;4:131–8. 12563284 10.1038/nrn1035

[16] Greig NH, Utsuki T, Yu Q-S, et al A new therapeutic target in Alzheimer's disease treatment: attention to butyrylcholinesterase. Curr Med Res Opin 2001;17:159–65. 11900310 10.1185/0300799039117057

[17] Recanatini M, Cavalli A. Acetylcholinesterase inhibitors in the context of therapeutic strategies to combat Alzheimer’s disease. Expert Opin Ther Pat 2005;12:1853–65.

[18] Ismaili L, Refouvelet B, Benchekroun M, et al Multitarget compounds bearing tacrine- and donepezil-like structural and functional motifs for the potential treatment of Alzheimer's disease. Prog Neurobiol 2017;151:4–34. 26797191 10.1016/j.pneurobio.2015.12.003

[19] Guzior N, Wieckowska A, Panek D, Malawska DB. Recent development of multifunctional agents as potential drug candidates for the treatment of Alzheimer’s disease. Curr Med Chem 2015;22:373–404. 25386820 10.2174/0929867321666141106122628 PMC4435057

[20] Bacalhau P, San Juan AA, Goth A, et al Insights into (S)-rivastigmine inhibition of butyrylcholinesterase (Buche): molecular docking and saturation transfer difference NMR (STD-NMR). Bioorg Chem 2016; 67:105–9. 27317888 10.1016/j.bioorg.2016.06.002

[21] Singh M, Silakari O. Design, synthesis and biological evaluation of novel 2-phenyl-1-benzopyran-4-one derivatives as potential poly-functional anti-Alzheimer's agents. RSC Adv 2016;6:108411–22.

[22] Li SY, Wang XB, Xie SS, et al Multifunctional tacrine-flavonoid hybrids with cholinergic, beta-amyloid-reducing, and metal chelating properties for the treatment of Alzheimer's disease. Eur J Med Chem 2013;69:632–46. 24095756 10.1016/j.ejmech.2013.09.024

[23] Sun Y, Chen J, Chen X, et al Inhibition of cholinesterase and monoamine oxidase-B activity by tacrine-homoisoflavonoid hybrids. Bioorg Med Chem 2013;21:7406–17. 24128814 10.1016/j.bmc.2013.09.050

[24] Desideri N, Bolasco A, Fioravanti R, et al Homoisoflavonoids: natural scaffolds with potent and selective monoamine oxidase-B inhibition properties. J Med Chem 2011;54:2155–64. 21405131 10.1021/jm1013709

[25] Liao S, Deng H, Huang S, et al Design, synthesis and evaluation of novel 5,6,7-trimethoxyflavone-6-chlorotacrine hybrids as potential multifunctional agents for the treatment of Alzheimer's disease. Bioorg Med Chem Lett 2015;25:1541–5. 25724825 10.1016/j.bmcl.2015.02.015

[26] Sang Z, Qiang X, Li Y, et al Design, synthesis and evaluation of scutellarein-O-alkylamines as multifunctional agents for the treatment of Alzheimer's disease. Eur J Med Chem 2015;94:348–66. 25778991 10.1016/j.ejmech.2015.02.063

[27] Ellman GL, Courtney KD, Andres V, Featherstone RM. A new and rapid colorimetric determination of acetylcholinesterase activity. Biochem Pharmacol 1961;7:88–95. 13726518 10.1016/0006-2952(61)90145-9

[28] Kim DH, Hung TM, Bae KH, et al Gomisin A improves scopolamine-induced memory impairment in mice. Eur J Pharmacol 2006; 542: 129–35. 16824513 10.1016/j.ejphar.2006.06.015

[29] Lima JA, Costa RS, Epifanio RA, et al Geissospermum vellosii stembark: anticholinesterase activity and improvement of scopolamine-induced memory deficits. Pharmacol Biochem Behav 2009;92:508–13. 19463267 10.1016/j.pbb.2009.01.024

[30] Kurt BZ, Gazioglu I, Basile L, et al Potential of aryl-urea-benzofuranylthiazoles hybrids as multitasking agents in Alzheimer's disease. Eur J Med Chem 2015;102:80–92. 26244990 10.1016/j.ejmech.2015.07.005

[31] Bourne Y, Sharpless KB, Taylor P, Marchot PP. Steric and dynamic parameters influencing in situ cycloadditions to form triazole inhibitors with crystalline acetylcholinesterase. J Am Chem Soc 2016;138:1611–21. 26731630 10.1021/jacs.5b11384

[32] Nachon F, Carletti E, Ronco C, et al Crystal structures of human cholinesterases in complex with huprine w and tacrine: elements of specificity for anti-Alzheimer's drugs targeting acetyl- and butyryl-cholinesterase. Biochem J 2013;453:393–9. 23679855 10.1042/BJ20130013

[33] Delcanale M, Amari G, Armani E, et al Novel basic isoflavones as inhibitors of bone resorption. Helv Chim Acta 2001;84:2417–29.

[34] Seguin H, Gardette D, Moreau M-F, et al A general method for the synthesis of N, N-dialkylaminobutylamines. Synth Commun 1998;28:4257–72.

[35] Overk CR, Felder CC, Tu Y, et al Cortical M1 receptor concentration increases without a concomitant change in function in Alzheimer’s disease. J Chem Neuroanat 2010;40:63–70. 20347961 10.1016/j.jchemneu.2010.03.005 PMC2864794

[36] Inesi A, Mucciante L, Rossi VL. A convenient method for the synthesis of carbamate esters from amines and tetraethylammonium hydrogen carbonate. J Organ Chem 1998;63:1337–8.

