# Supplementary material for: Discovery of novel isoflavone derivatives as AChE/BuChE dual-targeted inhibitors: synthesis, biological evaluation and molecular modelling
Source: J Enzyme Inhib Med Chem. 2017 Jul 18;32(1):968–77. doi: 10.1080/14756366.2017.1347163 (PMC6446070; doi:10.1080/14756366.2017.1347163)
Supplement: IENZ_1347163_Supplementary_Material.pdf [file IENZ_A_1347163_SM5073.pdf]

SUPPORTING INFORMATION

Discovery of novel isoflavone derivatives as AChE/BuChE dual-targeted inhibitors: synthesis, biological evaluation and molecular modeling

Bo Feng[1,#], Xinpeng Li[2,#], Jie Xia[1,*], Song Wu[1,*]

[1] State Key Laboratory of Bioactive Substance and Function of Natural Medicines, Department of New Drug Research and Development, Institute of Materia Medica, Chinese Academy of Medical Sciences and Peking Union Medical College, Beijing 100050, China; [2] Food and Drug Administration of Beijing Yanqing District, Beijing 102100, China

[#] These authors contributed equally to this work.

[*]Correspondence should be addressed to J.X. (jiexia@imm.ac.cn) or S.W. (ws@imm.ac.cn)

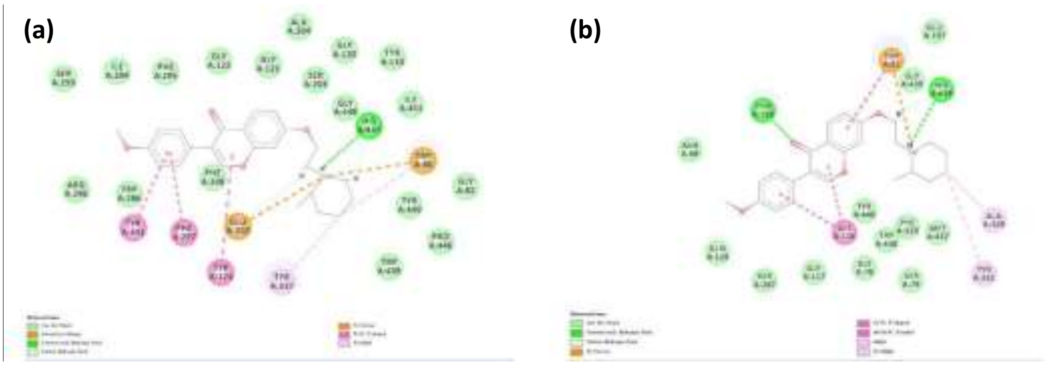

Figure S1. (a) 2D schematic diagram of potential interactions between compound **G** and AChE. (b) 2D schematic diagram of potential interactions between compound **G** and BuChE.
